# Efficacy of Web-Based, Guided Self-help Cognitive Behavioral Therapy–Enhanced for Binge Eating Disorder: Randomized Controlled Trial

**DOI:** 10.2196/40472

**Published:** 2023-05-01

**Authors:** Bernou Melisse, Elske van den Berg, Margo de Jonge, Matthijs Blankers, Eric van Furth, Jack Dekker, Edwin de Beurs

**Affiliations:** 1 Novarum Center for Eating Disorders Amstelveen Netherlands; 2 Section Clinical Psychology Leiden University Leiden Netherlands; 3 Research Department, Arkin Mental Health Institute Amsterdam Netherlands; 4 Trimbos Institute, Netherlands Institute of Mental Health and Addiction Utrecht Netherlands; 5 Department of Psychiatry, Location AMC, Amsterdam UMC, University of Amsterdam Amsterdam Netherlands; 6 Department of Psychiatry, Leiden University Medical Center Leiden Netherlands

**Keywords:** randomized controlled trial, RCT, binge eating disorder, BED, guided self-help, cognitive behavioral therapy–enhanced, CBT-E

## Abstract

**Background:**

Owing to the gap between treatment supply and demand, there are long waiting periods for patients with binge eating disorder, and there is an urgent need to increase their access to specialized treatment. Guided self-help cognitive behavioral therapy–enhanced (CBT-E) may have great advantages for patients if its efficacy can be established.

**Objective:**

The aim of this study was to examine the efficacy of guided self-help CBT-E compared with that of a delayed-treatment control condition.

**Methods:**

A single-blind 2-arm randomized controlled trial was designed to evaluate guided self-help CBT-E according to an intention-to-treat analysis. A total of 180 patients were randomly assigned to guided self-help CBT-E (n=90, 50%) or the delayed-treatment control condition (n=90, 50%) for which guided self-help CBT-E was provided after the initial 12-week delay. The primary outcome was reduction in binges. The secondary outcome was full recovery at the end of treatment, as measured using the Eating Disorder Examination during the last 4 weeks of treatment. A linear mixed model analysis was performed to compare treatment outcomes at the end of treatment. A second linear mixed model analysis was performed to measure between- and within-group effects for up to 24 weeks of follow-up. The Eating Disorder Examination–Questionnaire and clinical impairment assessment were conducted before and after treatment and during follow-up. In addition, dropout rates were assessed in both conditions.

**Results:**

During the last 4 weeks of treatment, objective binges reduced from an average of 19 (SD 16) to 3 (SD 5) binges, and 40% (36/90) showed full recovery in the guided self-help CBT-E group. Between-group effect size (Cohen *d*) was 1.0 for objective binges. At follow-up, after both groups received treatment, there was no longer a difference between the groups. Of the 180 participants, 142 (78.9%) completed treatment. The overall treatment dropout appeared to be associated with gender, level of education, and number of objective binges at baseline but not with treatment condition.

**Conclusions:**

This is the first study to investigate the efficacy of guided self-help CBT-E. Guided self-help CBT-E appeared to be an efficacious treatment. This study’s findings underscore the international guidelines recommending this type of treatment for binge eating disorder.

**Trial Registration:**

Netherlands Trial Registry (NTR) NL7994; https://trialsearch.who.int/Trial2.aspx?TrialID=NL7994

**International Registered Report Identifier (IRRID):**

RR2-10.1186/s12888-020-02604-1

## Introduction

### Background

Binge eating disorder (BED), recently included in the Diagnostic and Statistical Manual of Mental Disorders, fifth edition (DSM-5), is characterized by recurrent episodes of binge eating. The binges are accompanied by a sense of lack of control and feelings of shame, guilt, and disgust. However, the binges are not followed by inadequate compensatory behavior [1,2]. BED is the most common eating disorder and has an estimated lifetime prevalence of 2% [3] and up to 30% among people with excess weight [4]. BED has a substantial impact on the psychosocial functioning of individuals, affecting their personal, social, and cognitive domains [5]. Recently, the estimated prevalence of BED has increased, and patients seeking help display more severe symptoms, which is possibly related to the COVID-19 pandemic [6]. Around 33% to 48% of the patients reported increased eating disorder symptomatology [7,8]. Potential reasons for this increase during the pandemic are social isolation and decreased social support [9]. Other potential reasons include increased stress, restricted access to health care, and food insecurity [10]. Finally, increased social media exposure resulted in increased exposure to the thin ideal [11] and an uptick in fat-phobic messages, which lead to dieting behavior [10] and therefore an increase in binges [12].

Cognitive behavioral therapy–enhanced (CBT-E) is a recommended treatment for BED [12-14] and has remission rates of 50% to 68% in efficacy trials [15,16]. International guidelines recommend guided self-help based on cognitive behavioral principles for BED [17-19]. Only a few studies have examined the efficacy of guided self-help interventions for patients with BED [20,21]. Guided self-help studies based on regular cognitive behavioral therapy (CBT) report abstinence from binge eating after treatment among 46% of the participants and a sizable reduction in eating disorder pathology of a medium effect size [22,23]. However, the efficacy of web-based, guided self-help CBT-E has not yet been investigated.

Owing to the lack of specialized therapists in the Netherlands, as in many parts of the world, there is a gap between treatment supply and demand [24], resulting in long waiting periods for patients with BED. Therefore, there is an urgent need to increase access to treatment [25]. This situation worsened during the COVID-19 pandemic, when waiting times for treatment increased further and access to care decreased [26]. A remotely offered guided self-help version of CBT-E has the potential to offer treatment with reduced therapist involvement [27]. This, in turn, will enhance treatment availability and thus potentially reduce waiting time before treatment can commence, because long waiting times are unfavorable and associated with a negative treatment outcome [28].

Guided self-help CBT-E has advantages for the patient, such as the removal of geographical barriers and reduced travel costs and time, as communication with the therapist is enabled regardless of location [25,29-31]. However, there are potentially some disadvantages, such as higher attrition rates, less adherence, and a less credible image in both patients and therapists [32-34].

### Objective

The aim of this study was to examine the efficacy of guided self-help CBT-E compared with that of a delayed-treatment control condition through a randomized controlled trial (RCT) in patients with BED. The primary outcome was reduction in binge eating episodes, and the secondary outcome was the full recovery rate after treatment, as measured during the last 4 weeks of treatment. Web-based, guided self-help CBT-E was hypothesized to be superior to the control condition in reducing binge eating episodes and achieving full recovery. Follow-up measures will be conducted to measure the persistence of treatment benefits. It was hypothesized that treatment gains persist during the 12-week and 24-week follow-up and that there would be no differences between the groups after both groups received treatment.

## Methods

### Trial Design

A superiority RCT to examine the efficacy of web-based, guided self-help CBT-E at end of treatment (EOT) among patients with BED or other specified feeding or eating disorder (OSFED)–BED. Parallel groups were randomly assigned to one of two conditions as follows: (1) guided self-help CBT-E (n=89) or (2) a delayed-treatment control condition (n=91), in which guided self-help CBT-E was offered after a waiting period of 12 weeks. The assessors were blinded to the randomization. In addition, allocation was balanced (1:1) and randomization was stratified for BMI <29.9 kg/m^2^ or >30 kg/m^2^. The guided self-help CBT-E group was assessed at baseline (T0: week 0), week 5 (T1: intermediate evaluation of treatment), week 12 (T2: after treatment), week 24 (T3: 12-week follow-up), and week 36 (T4: 24-week follow-up). The delayed-treatment control group was assessed at baseline (T0: week 0), week 5 (T1: during waiting time), week 12 (T2: start of delayed treatment), week 24 (T3: after treatment), and week 36 (T4: 12-week follow-up). The study was performed in line with the updated CONSORT (Consolidated Standards of Reporting Trials) guidelines for reporting parallel group randomized trials [35].

The study was registered at the Dutch Trial Registry (NTR 7994). Details of the study have been published in the study protocol [36]. Study approval was given in August 2019 (reference number NL 6958.100.19) by the Medical Research Ethics Committees United.

### Participants

Eligible patients were aged ≥18 years, with a DSM-5 BED or OSFED-BED diagnosis [1], and had a BMI between 19.5 kg/m^2^ and 40 kg/m^2^, because CBT-E was explicitly designed for patients who were not underweight with a BMI of ≤40 kg/m^2^ [12]. Sufficient proficiency in Dutch and internet access were required. Exclusion criteria were eating disorders other than BED or OSFED-BED, acute psychosis, clinical depression or suicidal ideation, having received eating disorder treatment in the past 6 months, pregnancy, and use of medication that might influence eating behavior. For example, mirtazapine, olanzapine, clozapine, quetiapine, trazodone, and lithium increase appetite, whereas medications including methylphenidate and dexamphetamine decrease appetite [37]. The Dutch version of the semistructured interview the Structured Clinical Interview for DSM-5, Clinician Version (SCID-5-CV), assessing DSM-5 diagnoses [1,38], was used to establish the presence of diagnostic exclusion criteria. The interview sections for mood disorders and psychotic disorders were administered. The study was conducted at Novarum, the Dutch Eating Disorders and Obesity Department of Arkin, a large mental health care provider in Amsterdam. All eligible potential participants received verbal and written study information during an advisory session, including an informed consent description, explaining the research goals and information about participation. After patients provided informed consent, a baseline assessment (T0) was scheduled. Recruitment took place between September 2019 and October 2020. Diagnostic interviews were held in person until March 15, 2020, after which, because of the COVID-19 social distancing measures, all interviews were held through videoconferencing.

### Intervention

#### Overview

Treatment was offered by therapists with various backgrounds and educational levels (bachelor’s degree for dieticians and nurse practitioners; master’s and postdoctoral degree for psychologists). All therapists successfully completed a web-based CBT-E training provided by the Centre for Research on Eating Disorders at Oxford, United Kingdom. They first familiarized themselves with the detailed CBT-E manual and the guided self-help CBT-E manual [12]. They also attended a 2-day workshop provided by authors BM and MdJ. To ensure treatment adherence, all therapists attended weekly 45-minute supervision sessions with BM and rated their level of adherence after each session on a scale ranging from 0 (*not at all*) to 5 (*excellent*). Self-rated therapist adherence was very good, with 94.7% (1662/1755) of all sessions obtaining a maximum score for excellent adherence.

#### Guided Self-help CBT-E Condition

Guided self-help CBT-E started in the same week as the baseline assessment. Before commencing treatment, patients were required to read the psychoeducational section of the Dutch version of *Overcoming Binge Eating, The Proven Program to Learn Why You Binge and How You Can Stop.* Guided self-help CBT-E is a translated and digitalized version of part 2 of the self-help book *Overcoming Binge Eating* [39]*.* The intervention included psychoeducation, daily assignments, and 2 self-evaluations each week. When patients did not complete their daily assignments, they received reminders. Patients uploaded their assignments to the web-based therapy environment. Therapists were able to track when the patients logged in, read the psychoeducational parts, and started assignments. Once the patients completed their homework assignments, the therapist received a notification. Subsequently, feedback on the assignments was provided by the therapists during a weekly telephone session of 20 minutes. In the telephone session, completed assignments were discussed, as well as upcoming assignments and compliance with treatment. The sessions were scripted in accordance with the treatment manual as developed by EvdB and BM and offered by therapists through the telephone.

Similar to CBT-E–guided self-help, CBT-E consisted of 4 phases; the first stage focused on establishing regular eating and alternatives for binge eating; using real-time self-monitoring as the central intervention; and events, moods, and eating. After a joint review of progress and designing the rest of treatment in the second stage, based on the patients’ reported symptoms and maintaining mechanisms of their BED, the third stage focused on either dietary restraint or shape concern and finally ended well with a firm focus on minimizing the risk of relapse in the long term.

#### Delayed-Treatment Control Condition

Participants assigned to the delayed-treatment control condition started guided self-help CBT-E 12 weeks after baseline. Thus, their treatment started after a waiting period of the same duration as that of the intervention. Similar to the experimental condition, patients randomized to the control condition were advised to read the psychoeducational section of *Overcoming Binge Eating, The Proven Program to Learn Why You Binge and How You Can Stop* [39] before commencing treatment. This was recommended to bridge the 12-week waiting period and keep them involved and enrolled in the study. However, these patients did not receive any treatment assignments during this period and did not have access to the web-based treatment environment. Participants were called once after 6 weeks for a short conversation of 10 minutes at most: checking on the eating disorder symptoms and other important areas of life and answering questions about the recommended reading assignment.

### Outcomes

The primary outcome indicator was reduction in binge eating at T2. Binge eating was measured during the last 28 days using the Dutch Eating Disorder Examination (EDE), a validated expert interview tool. The secondary outcome indicator was full recovery at T2, which was defined as an EDE global score <1.77 as well as abstinence from binge eating during the last 28 days [40]. The cutoff on the EDE global score of <1.77 was based on the community mean plus 1 SD [41,42]. Other outcome measures were reliable change index (RCI) and clinically significant change (CSC) [43,44]. RCI was established as RCI=0.54 on the EDE global score, and CSC was defined as EDE global score <1.77 as well as a pre- to posttest change >RCI [41,43]. Outcome measures on self-report data were reduction of binge eating during the last 4 weeks measured at T2, T3, and T4 with the Dutch version of the EDE-Questionnaire (EDE-Q), a validated self-report questionnaire [45,46]. Full recovery was defined as an EDE-Q global score <2.77 (based on the community mean plus 1 SD) combined with the absence of binges, as described in Turner et al [40,47,48]. Cutoff on the EDE-Q was 2.77 and RCI was 0.63 on the EDE-Q global score, together they defined CSC [43,45]. The last outcome measure was the reduction of secondary impairment from eating disorder behavior during the last 28 days, as measured by the clinical impairment assessment (CIA) [5]. Interview data (EDE) were collected at baseline and after the conclusion of guided self-help CBT-E in the experimental group (T0 and T2). Data from self-report measures (EDE-Q and CIA) were collected at T0, T2, T3, and T4. In addition, the EDE-Q was also completed at T1, 5 weeks after treatment commenced, to evaluate treatment progression between the patient and therapist. Interviews were conducted by phone, and self-report measures were administered on the web. All assessments were processed using Castor EDC [49] (International Organization for Standardization [ISO]; ISO 27001/27002/9001 and NEN 7510 certified).

### Sample Size Estimation

On the basis of other self-help interventions, a 46% decrease in binge eating behavior was expected over time [22]. The expected effect size was a Cohen *d* of 0.47 between the experimental and control conditions [22,50]. To achieve sufficient power (β=.8), the required sample size was 144 (n=72 per arm). As a 20% dropout was estimated [22], more participants were included: N=180 (n=90 per arm), resulting in n=72 expected completers, yielding a power of β=.8, with an effect size of Cohen *d*=0.47, at α=.05 (2-sided). Sample size was calculated using R package (R Foundation for Statistical Computing) *pwr* [51].

### Randomization and Blinding

Randomizations were performed by administrative staff members of another department in Castor EDC [49] by a 4, 6, 8 block design. Assessors were research assistants with a master’s degree in psychology who were blinded to the allocated treatment condition, as were the staff members performing randomizations. In addition, when offering treatment, therapists were not aware of whether patients had previously been allocated to the experimental or control condition.

### Statistical Analysis

#### Baseline Differences

The significance of baseline differences between the groups was examined using chi-square tests or ANOVA.

#### Treatment Adherence

Regression analyses were conducted to assess whether baseline scores (number of objective binges, eating disorder severity, and BMI) and demographics (age, gender, level of education, profession, and country of birth) predicted treatment completion.

#### 2 × 2 Design

The primary outcome was treatment effects based on interview data (EDE) with regard to reduction in binge eating episodes and full recovery at posttest between the experimental and delayed-treatment control group, which were compared after 12 weeks, when the experimental group had concluded treatment (T2). As patients were initially supposed to be nested within their BMI group as described in the protocol [36], for the primary outcome measures, a 2 × 2 design was used using a generalized linear mixed model analysis [52], with group as the between-subjects factor and time of assessment as the within-subjects factor at the primary end point. As full recovery was a binary variable, a negative binomial model with log link was used.

#### 2 × 5 Design

Self-report data (EDE-Q and CIA) were analyzed with a 2 × 5 generalized linear mixed model analysis [52], with group as the between-subjects factor and time of assessment as the within-subjects factor, which also measured persistence of treatment benefits after EOT. For full recovery (binary variable), we used a negative binomial model with log link.

#### Effect Sizes

Effect sizes for both designs were calculated between and within groups using Cohen *d* (0.2, small; 0.5, medium; and 0.8, large) [50].

#### Imputation and Software

Analyses were performed according to an intention-to-treat approach (imputed data set with 25 imputations for each missing observation) [53]. Imputations were performed with the multiple imputation by chained equations, using predictive mean matching combining 25 imputations in R package *mice* [54]. All other statistical analyses were performed using SPSS (IBM Corp) versions 25 and 28.

### Ethics Approval and Informed Consent

Study approval (reference number NL 6958.100.19) was granted in August 2019 by the Medical Research Ethics Committees United in Nieuwegein, the Netherlands. All patients were informed about the study and assured that their data were deidentified, and all patients signed an informed consent form.

## Results

### Patient Flow

**Figure 1:**
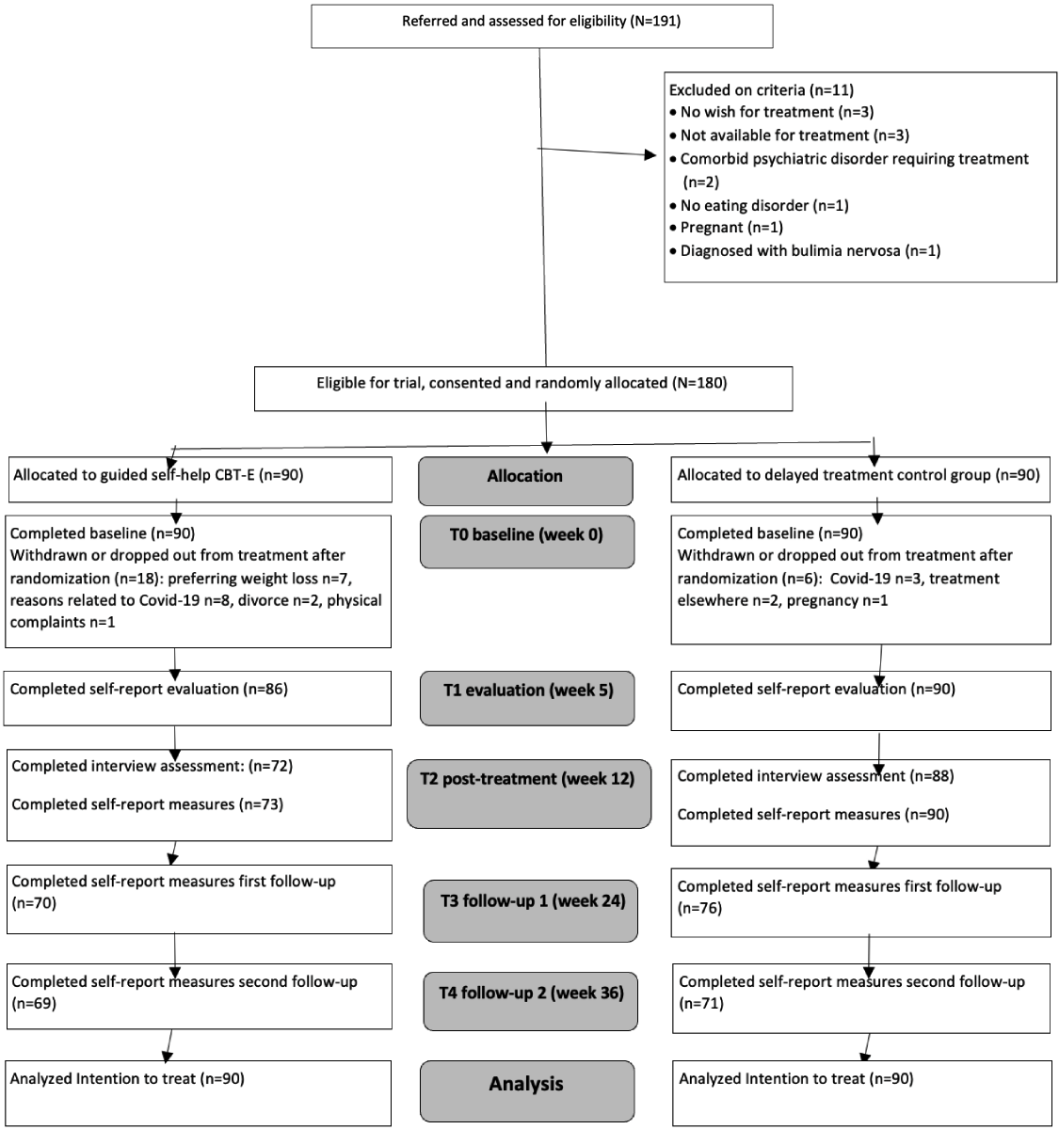
Flowchart of patients in the study. CBT-E: cognitive behavioral therapy–enhanced; T0: assessment week 0; T1: assessment week 5; T2: assessment week 12; T3: assessment week 24; T4: assessment week 36.

Potential participants (N=191) were recruited between September 2019 and October 2020. In total, 180 patients were randomized, excluding 11 who did not meet the inclusion criteria or met the exclusion criteria; 176 were diagnosed with BED of which 4 had a history of bariatric surgery, had smaller binges, and were therefore diagnosed with OSFED-BED. The CONSORT flow diagram (Figure 1) shows participant enrollment and flow throughout the study, and Table 1 summarizes participant characteristics at baseline. The treatment conditions were comparable; there were no significant differences between the 2 conditions (*P*>.05). One patient withdrew before the baseline assessment was completed. Last therapy concluded in April 2021, and last follow-up data were completed in August 2021. No serious adverse events occurred during the trial.

**Table 1:** Patient characteristics at baseline.

Characteristics		Total sample (N=180)	Experimental condition (n=90)	Delayed-treatment control group (n=90)	*P* value	
Age (years), mean (SD)		39.4 (13.1)	39.2 (13.6)	40.6 (13.5)	.76	
Baseline BMI (kg/m^2^), mean (SD)		33.4 (5.3)	34.0 (5.6)	32.9 (5.0)	.51	
**Gender, n (%)**						.55
	Women	163 (90.6)	83 (92.1)	80 (90.9)		
	Men	17 (9.4)	7 (9.2)	10 (11.3)		
**Highest level of education, n (%)**						.61
	No education	0 (0)	0 (0)	0 (0)		
	Primary school	0 (0)	0 (0)	0 (0)		
	Lower vocational education	5 (2.8)	4 (4.9)	1 (1.2)		
	Lower general secondary education	7 (3.9)	5 (6.6)	2 (2.5)		
	Senior general secondary education or university preparatory education	15 (8.3)	5 (6.6)	10 (12.3)		
	Secondary vocational education	51 (28.3)	23 (26.2)	27 (29.6)		
	Higher professional education	63 (35)	33 (37.7)	30 (34.6)		
	University	35 (21.1)	16 (19)	19 (21)		
	Unknown	1 (0.6)	0 (0)	1 (1.2)		
**Profession, n (%)**						.051
	Student	19 (10.6)	9 (9.8)	10 (12.3)		
	Employed	120 (66.7)	55 (63.9)	65 (74.1)		
	Volunteer job	6 (3.3)	4 (4.9)	2 (2.5)		
	Unemployed	12 (6.7)	1 (1.6)	8 (8.6)		
	Other	23 (12.8)	17 (19.7)	6 (6.2)		
**Civil status, n (%)**						.99
	Single	101 (56.6)	45 (50.8)	48 (53.1)		
	Registered partnership	12 (6.7)	6 (8.2)	6 (7.4)		
	Married	56 (31.1)	31 (34.4)	29 (32.1)		
	Divorced	11 (6.1)	5 (6.6)	6 (7.4)		
Duration of eating disorder (years), mean (SD)		25.04 (4.15)	23.07 (3.85)	26.23 (4.36)	.37	
**Eating disorder treatment in the past, n (%)**						.49
	Yes	30 (16.7)	14 (16.4)	19 (21)		
	No	150 (83.3)	74 (83.6)	71 (79)		
**Comorbid diagnosis, n (%)**						.77
	No	77 (42.2)	33 (37.7)	44 (44.4)		
	I do not know	25 (13.9)	14 (18)	11 (13.9)		
	Mood disorder	24 (13.3)	10 (11.5)	14 (16)		
	Anxiety disorder	11 (6.1)	7 (9.8)	4 (4.9)		
	Attention-deficit/hyperactivity disorder	11 (6.1)	5 (6.6)	6 (7.4)		
	Posttraumatic stress disorder	6 (3.3)	5 (6.6)	1 (1.2)		
	Personality disorder	11 (6.1)	9 (9.8)	2 (2.5)		
	Autism	6 (3.3)	6 (8.2)	0 (0)		
	Other	15 (8.3)	6 (8.2)	9 (11.1)		
**Use of medication, n (%)**						.59
	Yes	45 (25.6)	23 (27.9)	22 (25.9)		
	No	134 (74.4)	64 (72.1)	67 (74.1)		
**Eating disorder pathology (EDE^a^), mean (SD)**						
	Total score	3.03 (0.9)	3.4 (1.0)	3.0 (0.9)	.49	
	Dietary restraint	2.2 (1.2)	2.9 (1.1)	2.0 (1.3)	.09	
	Eating concern	2.5 (1.3)	3.5 (1.3)	2.3 (1.2)	.60	
	Weight concern	3.6 (1.1)	3.6 (1.1)	3.6 (1.1)	.85	
	Shape concern	3.8 (1.2)	3.8 (1.3)	3.8 (1.1)	.67	
Eating disorder pathology (EDE-Q^b^ total score), mean (SD)		3.5 (1.0)	3.9 (1.0)	3.5 (1.0)	.48	
**Binge eating (EDE),** **mean (SD)**						
	Objective episodes	17.9 (14.5)	19.4 (16.3)	16.0 (13.8)	.40	
	Subjective episodes	14.5 (20.2)	17.8 (25.6)	14.7 (17.9)	.11	
	Days with objective episodes	14.3 (8.8)	15.46 (8.8)	12.9 (8.1)	.31	
	Days with subjective episodes	9.4 (10.2)	11.1 (11.3)	9.7 (10.1)	.11	
**Secondary pathology (CIA^c^), mean (SD)**						
	Total score	22.3 (8.6)	23.21 (8.4)	22.0 (8.2)	.58	
	Personal	13.2 (4.2)	13.63 (3.7)	13.3 (4.0)	.49	
	Social	4.8 (2.7)	5.01 (2.6)	4.6 (2.8)	.72	
	Cognitive	4.3 (3.4)	4.55 (3.8)	4.7 (3.2)	.36	

^a^EDE: Eating Disorder Examination.

^b^EDE-Q: Eating Disorder Examination–Questionnaire.

^c^CIA: clinical impairment assessment.

### Treatment Adherence

Participants were considered completers once they attended 11 sessions. Of the participants who started treatment (N=180), 142 completed at least 11 sessions (overall completion rate: 142/180, 78.9%; experimental condition: 69/90, 78%; control condition: 73/90, 80%). As only 10.7% (19/180) of the participants had a BMI <30 kg/m^2^, no subgroup analyses based on stratification below and above BMI 30 kg/m^2^ were performed. Treatment dropout was higher among men (*χ*^2^_1_=7.6; *P*=.01), less-educated patients (*χ*^2^_5_=18.8; *P*=.005), and patients who displayed a greater number of objective binges at the start (t_178_=49.90; *P*=.02). Treatment completion was not predicted by treatment condition (*P*=.54), age (*P*=.51), profession (*P*=.45), marital status (*P*=.18), eating disorder treatment in the past (*P*=.27), medication use (*P*=.47), BMI (*P*=.64), EDE restraint (*P*=.73), EDE eating (*P*=.38), EDE weight concern (*P*=.28), EDE shape concern (*P*=.19), and EDE global score (*P*=.21). Study dropout among participants who completed treatment was 2.8% (5/180), 1.7% (3/180) of patients did not complete the follow-up measures at T3 weeks and T4 weeks, and for 2.8% (2/180) of additional patients, no assessments at T4 were available.

### Outcomes

#### Binges

Table 2 shows that at EOT, as measured by the EDE, the guided self-help group had 3 objective binges during the last 28 days and the delayed-treatment group had 13 binges during the last 28 days of their wait time. At T2, in total, 48% (42/90) of the participants assigned to the guided self-help CBT-E showed abstinence of binge eating during the last 4 weeks. A 2 × 2 generalized linear mixed model analysis with fixed effects showed differences between the experimental and control groups at T2. There was an interaction effect between time and treatment condition (*F*_2,178_=18.55; *P*<.001). Comparable results were found for subjective binges (*F*_2,178_=10.08; *P*<.001). When the same analysis was repeated for objective binges as measured by the EDE-Q, a 2 × 5 generalized linear mixed model analysis with fixed effects showed an interaction effect between time and treatment condition (*F*_7,173_=108.82; *P*<.001). However, the difference disappeared when both groups received treatment at T3 (*P*=.59) and T4 (*P*=.69). Results from both analyses indicated that objective binges reduced faster in the guided self-help group than in the delayed-treatment group. Assessments at T3 and T4 showed persistence of treatment benefits for patients of the experimental condition. There were no differences between the intention-to-treat and the completers sample.

**Table 2:** Changes in binge eating behaviors and Eating Disorder Examination (EDE) scores over the course of treatment assessed using intention-to-treat analysis with multiple imputations.

	Guided self-help CBT-E^a^ (n=90)			Within groups T0^b^-T2^c^, EMD^d^ (95% CI)	Within groups T0-T2 (effect size), Cohen *d* (95% CI)	Delayed-treatment control condition (n=90)					Within groups T0-T2 (effect size), Cohen *d* (95% CI)		Between groups at T2, EMD (95% CI)		Effect size, Cohen *d*
	T0, mean (SD)	T2, mean (SD)	*F* test (*df*)			T0, mean (SD)	T2, mean (SD)	*F* test (*df*)	Within groups T0-T2, EMD (95% CI)						
Number of objective binges	19.4 (16.3)	2.6 (5.2)	78.9^e^ (1,178)	−16.8 (−20.4 to −13.2)	1.4 (1.1 to 1.7)	16.0 (13.8)	13.1 (13.8)	4.3	−3.0 (−1.0 to 7.0)	0.2 (−0.1 to 0.5)		−10.4 (−13.6 to −7.3)		1.0	
Days objective binges	15.5 (8.8)	2.2 (3.5)	121.7^e^ (1,178)	−13.3 (−15.2 to −11.3]	2.0 (1.6 to 2.3)	12.9 (8.1)	10.3 (8.1)	7.6	−2.6 (−5.0 to −0.3)	0.3 (0.0 to 0.6)		−8.1 (−9.9 to −6.2)		1.3	
Number of subjective binges	17.8 (25.6)	4.7 (8.8)	13.7^e^ (1,178)	−13.1 (−18.8 to −7.4)	0.7 (0.4 to 1.0)	14.7 (17.9)	14.9 (24.1)	0.1	−0.8 (6.4 to 6.0)	0.0 (−0.3 to 0.3)		−10.3 (−15.6 to −4.9)		0.6	
Days of subjective binges	11.1 (11.3)	4.0 (5.9)	19.5^e^ (1,178)	−7.1 (−9.8 to −4.4)	0.8 (0.5 to 1.1)	9.7 (10.1)	9.9 (10.5)	0.0	0.0 (−3.1 to 3.0)	0.0 (−0.3 to 0.3)		−5.9 (−8.4 to −3.4)		0.7	
EDE global score	3.4 (1.0)	1.7 (0.9)	125.8^e^ (1,178)	−1.7 (−2.0 to −1.4)	1.8 (1.4 to 2.1)	3.0 (0.9)	2.8 (0.9)	3.6	−0.2 (−0.1 to 0.4)	0.2 (−0.1 to 0.5)		−1.1 (−1.4 to −0.8)		1.2	
EDE dietary restraint	2.9 (1.1)	0.7 (0.9)	106^e^ (1,178)	−2.2 (−2.5 to −1.9)	2.1 (1.7 to 2.5)	2.0 (1.3)	1.6 (1.2)	5.4	−0.4 (−0.8 to 0.0)	0.3 (0.0 to 0.6)		−0.9 (−1.2 to −0.5)		0.8	
EDE eating concern	3.5 (1.3)	1.1 (1.0)	84.2^e^ (1,178)	−2.4 (−2.8 to −2.1)	2.1 (1.7 to 2.5)	2.3 (1.2)	2.3 (1.3)	0.1	0.1 (−0.4 to 0.4)	0.0 (−0.3 to 0.3)		−1.2 (−1.6 to −0.9)		1.1	
EDE shape concern	3.8 (1.3)	2.5 (1.2)	69.0^e^ (1,178)	−1.3 (−1.7 to −0.9)	1.0 (0.7 to 1.3)	3.8 (1.1)	3.8 (1.1)	0.5	−0.1 (−0.2 to 0.4)	0.0 (−0.2 to 0.6)		−1.3 (−1.6 to −0.9)		1.1	
EDE weight concern	3.6 (1.1)	2.5 (1.2)	56.0^e^ (1,178)	−1.2 (−1.5 to −0.8)	1.0 (0.7 to 1.3)	3.6 (1.1)	3.5 (1.1)	0.6	−0.1 (−0.2 to 0.4)	0.1 (−0.2 to 0.6)		−1.03 (−1.4 to −0.7)		0.9	

^a^CBT-E: cognitive behavioral therapy–enhanced.

^b^T0: assessment week 0.

^c^T2: assessment week 12.

^d^EMD: estimated mean difference.

^e^*P*<.001.

#### Full Recovery

As measured by the EDE, at EOT, full recovery was achieved in 40% (36/90) during the last 28 days in the guided self-help group and 7% (6/90) fully recovered during the last 28 days of their wait time (Table 3). A CSC was achieved by 56% (51/90) and 7% (6/90) in the experimental and control conditions, respectively. An interaction effect between time and treatment condition at T2 (*F*_2,178_=7.90, *P*=.006) was found in a 2 × 2 generalized linear mixed model analysis with fixed effects. This indicated greater recovery based on the EDE in the guided self-help CBT-E group than in the delayed-treatment group. A 2 × 5 analysis based on EDE-Q data showed an interaction effect between time and treatment condition (*F*_7,173_=14.02; *P*<.001). This difference disappeared when both groups received treatment at T3 (*P*=.99) and T4 (*P*=.99). Both results indicate that the guided self-help group recovered faster than the delayed-treatment group.

**Table 3:** Remission rates for the intention-to-treat sample.

				T0^a^, n (%)		T2^b^, n (%)		T3^c^, n (%)		T4^d^, n (%)
**Guided self-help CBT-E^e^ (n=90)**										
	**EDE^f^**							N/A^g^		N/A
		Absence of objective binges	5 (6)		43 (48)					
		EDE global<1.77	5 (6)		56 (62)					
		Full recovery^h^	0 (0)		36 (40)					
		RCI^i^	N/A		71 (79)					
		CSC^j,k^	N/A		51 (57)					
		Unchanged	N/A		5 (6)					
		Deteriorated	N/A		13 (15)					
		EDE restraint<1.75	27 (30)		74 (82)					
		EDE eating concern<0.86	7 (8)		49 (54)					
		EDE shape concern<2.43	16 (18)		48 (53)					
		EDE weight concern<2.11	4 (4)		36 (40)					
	**EDE-Q^l^**									
		Absence of objective binges	0 (0)		20 (22)		42 (47)		38 (42)	
		EDE-Q score<2.77	2 (2)		71 (79)		64 (71)		58 (64)	
		Full recovery^m^	0 (0)		19 (21)		35 (39)		32 (36)	
		RCI	N/A		71 (79)		70 (78)		65 (72)	
		CSC^n^	N/A		59 (66)		58 (64)		51 (57)	
		Unchanged	N/A		6 (7)		4 (5)		13 (15)	
		Deteriorated	N/A		3 (3)		6 (7)		6 (7)	
	CIA^o^<16		22 (24)		68 (76)		65 (72)		64 (71)	
**Delayed-treatment control group (n=90)**										
	**EDE**							N/A		N/A
		Absence of objective binges	3 (3)		9 (10)					
		EDE global<1.77	10 (11)		11 (12)					
		Full recovery^h^	1 (1)		6 (7)					
		RCI	N/A		21 (24)					
		CSC^k^	N/A		6 (7)					
		Unchanged	N/A		36 (40)					
		Deteriorated	N/A		11 (12)					
		EDE restraint<1.75	37 (41)		53 (58)					
		EDE eating concern<0.86	7 (8)		9 (10)					
		EDE shape concern<2.43	9 (10)		12 (13)					
		EDE weight concern<2.11	8 (9)		11 (12)					
	**EDE-Q**									
		Absence of objective binges	1 (1)		7 (8)		28 (31)		28 (31)	
		EDE-Q<2.77	20 (22)		29 (32)		69 (76)		58 (64)	
		Full recovery^m^	1 (1)		3 (3)		26 (29)		25 (28)	
		RCI	N/A		28 (31)		76 (84)		61 (68)	
		CSC^n^	N/A		19 (21)		65 (72)		52 (58)	
		Unchanged	N/A		40 (44)		10 (11)		11 (12)	
		Deteriorated	N/A		6 (7)		1 (1)		6 (7)	
	CIA<16		26 (29)		27 (30)		62 (69)		63 (70)	

^a^T0: assessment week 0.

^b^T2: assessment week 12.

^c^T3: assessment week 24.

^d^T4: assessment week 36.

^e^CBT-E: cognitive behavioral therapy–enhanced.

^f^EDE: Eating Disorder Examination (full recovery: Eating Disorder Examination<1.77, BMI>18.5 kg/m^2^ and no binge eating.).

^g^N/A: not applicable.

^h^Eating Disorder Examination<1.77, BMI>18.5 kg/m^2^ and no binge eating.

^i^RCI: reliable change index.

^j^CSC: clinically significant change.

^k^Combination of Eating Disorder Examination<1.77 and reliable change: reduction of 0.54 on the Eating Disorder Examination global score.

^l^EDE-Q: Eating Disorder Examination–Questionnaire.

^m^Eating Disorder Examination–Questionnaire<2.77, BMI>18.5 kg/m^2^ and no binge eating.

^n^Combination of Eating Disorder Examination–Questionnaire<2.77 and reliable change: reduction of 0.63 on the Eating Disorder Examination–Questionnaire global score.

^o^CIA: Clinical Impairment Assessment.

#### Global Scores on Eating Disorder Measures

**Figure 2:**
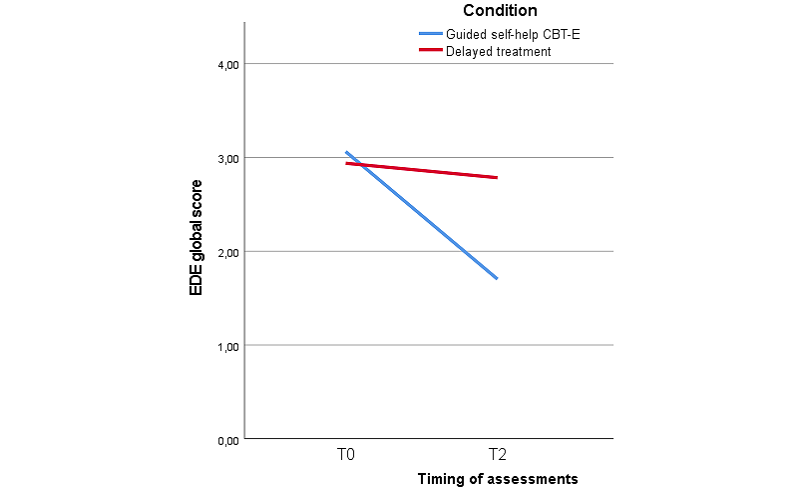
Mean Eating Disorder Examination (EDE) global scores of the intention-to-treat sample at T0 and T2. CBT-E: cognitive behavioral therapy–enhanced; T0: assessment week 0; T2: assessment week 12.

**Figure 3:**
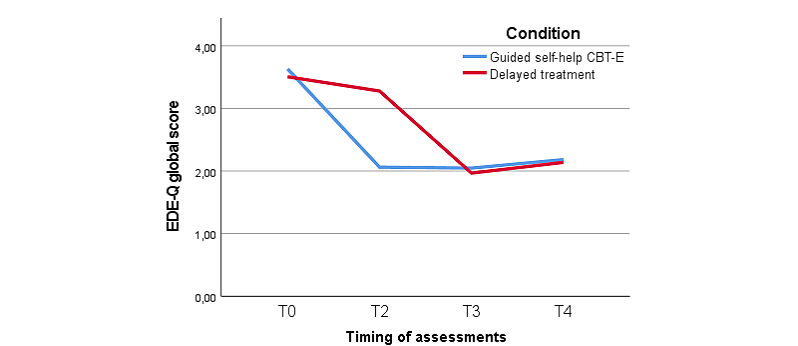
Mean Eating Disorder Examination–Questionnaire (EDE-Q) scores of the intention-to-treat sample at T0, T1, T2, T3, and during T4. CBT-E: cognitive behavioral therapy–enhanced; T0: assessment week 0; T1: assessment week 5; T2: assessment week 12; T3: assessment week 24; T4: assessment week 36.

Figure 2 shows that a 2 × 2 generalized linear mixed model analysis with fixed effects showed differences in the EDE global score between the experimental and control group at T2. An interaction effect between time and treatment condition at T2 (*F*_2,178_=73.50; *P*<.001) was found. This indicated that over time, patients in the guided self-help CBT-E condition had a greater reduction in their EDE scores than those in the control condition (Table 3). In addition, a 2 × 5 generalized linear mixed model analysis with fixed effects based on the EDE-Q global score showed an interaction effect between time and treatment condition (*F*_7,173_=42.65; *P*<.001). This difference disappeared when both groups received treatment at T3 (*P*=.52) and T4 (*P*=.31). Assessments at T3 and T4 showed the persistence of treatment benefits for patients in the experimental condition. Figure 3 and Table 4 show that patients randomized to the delayed-treatment control condition remained stable in the experimental phase of the trial (for them, the waiting period) but showed a delayed treatment effect very similar to the guided self-help group, consistent with the delayed design: eating disorder pathology decreased at T3 in the control condition and benefits persisted until T4.

**Table 4:** Changes in binge eating behaviors, Eating Disorder Examination–Questionnaire (EDE-Q) scores, BMI, and secondary eating disorder.

		Values, mean (SD)						*F* test (*df*)		Within groups (effect size), Cohen *d*					Between groups (effect size), Cohen *d*							
		T0^a^	T1^b^	T2^c^	T3^d^	T4^e^			T0-T1		T0-T2	T0-T3	T0-T4	T1				T2	T3			T4
**Guided self-help CBT-E^f^ (n=90)**																						
	EDE-Q Objective binges	15.8 (11.8)	7.7 (7.3)	3.4 (3.7)	3.4 (4.9)	3.2 (4.7)	21.6^g^ (1,178)		0.8		1.4	1.4	1.4	0.4				1.2	0.0			0.2
	EDE-Q global score	3.9 (1.0)	2.9 (0.9)	2.0 (1.0)	2.1 (1.2)	2.2 (1.3)	46.9^g^ (1,178)		1.0		1.9	1.7	1.5	0.5				1.3	0.1			0.1
	BMI (kg/m^2^)	34. (5.6)	34.4 (6.1)	35.4 (7.2)	33.9 (6.1)	33.9 (6.1)	0.8 (1,178)		0.1		−0.2	0.0	0.0	0.2				0.2	0.1			0.1
	CIA^h^ total score	23.2 (8.4)	N/A^i^	12.0 (8.8)	11.3 (9.2)	12.1 (9.8)	45.0^g^ (1,178)		N/A		1.3	1.4	1.2	N/A				1.1	0.2			0.0
	CIA personal	13.6 (3.7)	N/A	7.7 (4.3)	7.1 (4.6)	7.9 (5.3)	37.4^g^ (1,178)		N/A		1.5	1.5	1.3	N/A				1.1	0.3			0.1
	CIA social	5.0 (2.6)	N/A	2.1 (2.3)	2.0 (2.5)	2.2 (2.7)	31.5^g^ (1,178)		N/A		1.2	1.2	1.1	N/A				0.9	0.1			0.0
	CIA cognitive	4.6 (3.8)	N/A	2.2 (3.0)	2.2 (1.3)	2.1 (2.8)	19.2^g^ (1,178)		N/A		0.7	0.8	0.7	N/A				0.8	0.1			0.1
**Waiting list (n=90)**																N/A	N/A			N/A	N/A	
	EDE-Q objective binges	14.6 (10.1)	11.6 (7.7)	10.6 (8.1)	3.3 (4.4)	4.6 (7.0)	38.2^g^ (1,178)		0.3		0.4	1.5	1.2									
	EDE-Q global score	3.5 (1.0)	3.4 (0.9)	3.3 (1.0)	2.0 (1.1)	2.1 (1.3)	87.6^g^ (1,178)		0.1		0.3	1.5	1.2									
	BMI (kg/m^2^)	32.9 (5.0)	33.1 (7.2)	33.9 (8.8)	33.3 (4.9)	33.1 (4.9)	0.9 (1,178)		0.1		−0.2	−0.1	−0.1									
	CIA total score	22.0 (8.2)	N/A	21.5 (8.6)	13.0 (8.1)	12.2 (9.9)	40.9^g^ (1,178)		N/A		0.1	1.1	1.1									
	CIA personal	13.3 (4.0)	N/A	12.6 (4.4)	8.2 (4.1)	7.6 (5.2)	45.4^g^ (1,178)		N/A		0.2	1.3	1.2									
	CIA social	4.6 (2.8)	N/A	4.3 (2.7)	2.3 (2.0)	2.3 (2.7)	23.4^g^ (1,178)		N/A		0.1	1.0	0.9									
	CIA cognitive	4.1 (3.2)	N/A	4.6 (3.3)	2.5 (2.9)	2.3 (3.1)	17.4^g^ (1,178)		N/A		−0.2	0.5	0.6									

^a^T0: assessment week 0.

^b^T1: assessment week 5.

^c^T2: assessment week 12.

^d^T3: assessment week 24.

^e^T4: assessment week 36.

^f^CBT-E: cognitive behavioral therapy–enhanced.

^g^*P*<.001.

^h^CIA: clinical impairment assessment.

^i^N/A: not applicable.

#### Clinical Impairment

On the basis of CIA scores, there was an interaction effect between time and treatment (*F*_7,173_=90.36; *P*<.001). This indicated that over time, patients’ CIA scores reduced faster in the guided self-help CBT-E condition than in the control condition. The difference disappeared at T3 (*P*=.98) and T4 (*P*=.91), when both groups received treatment.

#### Effect Sizes

Table 2 shows large effect sizes between both conditions at T2 regarding objective binges (Cohen *d*=1.0-1.3) and EDE global score (Cohen *d*=1.2). Effect size was medium regarding subjective binges (Cohen *d*=0.6-0.7). Table 4 shows the effect sizes of the self-report measures.

## Discussion

### Principal Findings

The aim of this study was to examine the efficacy of guided self-help CBT-E compared with a delayed-treatment control group regarding reduction in objective binges. The efficacy of guided self-help CBT-E was demonstrated by its superiority in outcome over the delayed-treatment control condition at T2. On the basis of reduction in binge eating, a large effect size (Cohen *d*=1.0) was observed. Binge eating reduced from an average of 19 objective binges 28 days before assessment to 3 binges after completion of guided self-help CBT-E, compared with 16 to 13 binges in the control group. In the guided self-help condition, abstinence from binge eating at T2 was reported by 48% (43/90) of the participants according to the EDE interview.

Recovery rates for all other outcome measures were superior at T2 in the guided self-help condition than in the delayed-treatment control condition. In the guided self-help condition, 40% (36/90) of the participants showed full recovery according to the EDE interview, and eating disorder pathology score was below the clinical cutoff of 62% (56/90). Of them, 79% (71/90) reported an eating disorder pathology score below the clinical cutoff on self-report data. Follow-up data revealed no differences between the groups after both groups had received treatment. Treatment benefits persisted at T3 and T4 for the experimental condition and at T4 for the control condition. BMI did not change over the course of treatment, which can be interpreted as the prevention of weight gain.

Reduction in binges [15,55] and abstinence from binge rates [15,47,55,56] were comparable with in-person CBT-E at EOT and follow-up [22,56]. However, our study had larger effect sizes with regard to reduction in binges compared with that of in-person CBT-E [57,58]. It should be noted that owing to a lack of studies focusing on the BED populations specifically, comparisons of this study results with in-person CBT-E could mostly be made with samples of transdiagnostic patients or patients with bulimia. Moreover, the abstinence from binge rates in this study was comparable with other guided self-help interventions of regular CBT for BED at EOT and follow-up [22]. Furthermore, within-group effect sizes were large in this study but medium in studies examining the efficacy of regular CBT for BED [22,59,60]. Therefore, with regard to reduction in binges, it can be concluded that guided self-help CBT-E could be as effective as in-person CBT-E and other guided self-help interventions based on regular CBT.

The proportion of patients with eating disorder pathology scoring below the cutoff on the eating disorder measures indicated that guided self-help CBT-E is at least as effective as guided self-help interventions based on regular CBT [22,61]. Superiority based on the EDE in comparison with in-person CBT-E was inconclusive: Fairburn et al [15] showed greater remission, while efficacy in the studies by Poulsen et al [56] and Thompson-Brenner et al [62] was equal, but efficacy was lower in the study by Wonderlich et al [63]. In contrast, our study showed that guided self-help CBT-E appeared to be at least as effective at EOT, based on EDE-Q data [47,55,57,64,65]. RCI and CSC were larger in this study than in in-person CBT-E effectiveness studies [58,66].

We found that the severity of binge eating, eating disorder pathology, and secondary impairment in our study were comparable with those of previous studies that included patients with BED and transdiagnostic samples [15,20,47,58,67]. Therefore, the results of our study were not because of lower severity at baseline. However, it should be noted that guided self-help CBT-E was offered in a specialized eating disorder center. Enrolled patients had more severe BED compared with those from nonspecialist centers [68]. Furthermore, patients received guided self-help CBT-E from highly trained therapists, which might have affected the results. Therefore, these results may not be generalizable to nonspecialized settings. Further studies are needed to investigate the efficacy of the present treatment when delivered by less-specialized therapists to less severely ill patients.

Treatment dropout rate was 21.1% (38/180), and the majority dropped out during the COVID-19 pandemic (34/180, 89.5%), with one-third owing to reasons related to the COVID-19 pandemic. Treatment dropout rate was comparable with that of other studies, including a waiting-list control condition [22]. Patients with lower education had a higher chance of dropping out of the treatment. A negative attitude toward psychological treatment may have played a role, which might be reduced by offering psychoeducation [69]. Furthermore, these patients may have perceived some of the interventions as challenging, and extra assistance in overcoming such barriers may help keep them involved [70].

### Strengths and Limitations

This study has several strengths. It was conducted in a specialized mental health care setting, acknowledged for its highly structured treatment and evidence-based approach. Guided self-help CBT-E was a manualized treatment offered by trained specialists and treatment adherence was assessed. Standardized interview data [71] were collected by independent assessors, including the EDE at T2. Internationally used valid self-report instruments [5,45] were used, and the study was adequately powered. As patients came from all over the Netherlands, the sample can be deemed representative of patients seeking specialized eating disorder treatment. The COVID-19 pandemic deserves a special mention. The study barely started when the COVID-19 pandemic spread in the Netherlands in mid-March 2020. Fortunately, however, because of the treatment delivery mode (e–mental health) that was evaluated in this study, the social distancing measures of the pandemic had a limited impact on the study’s execution. Nevertheless, the COVID-19 pandemic might have negatively affected the outcomes of the treatments, as many patients reported that it was a challenge to combine therapy, work, and homeschooling children at the same time. This suggests that guided self-help CBT-E might demonstrate even better outcomes under less adverse circumstances.

A limitation of this study might be that the follow-up data were measured by self-report, and interview data are generally viewed as more reliable, especially when measuring binge eating behavior [72,73]. In addition, our study showed differences in reports on interviews and self-report data. Objective binges between the interview and self-report data in this study showed a moderate correlation (*r*=0.6; *P*<.001) at T2. The study’s design with a delayed-treatment control group implies that expected treatment benefits may have played a role in bringing about the difference in outcomes at the second assessment [74]. However, the extent of this effect could not be established, as treatment expectancy was not assessed. Next, between-group comparisons were impacted as the control group started treatment after the 12-week delay. Therefore, the long-term impact of withholding treatment could not be assessed. The control group showed a delayed treatment effect very similar to that of the guided self-help group, consistent with the delayed design. Furthermore, only within-group comparisons were meaningful during follow-up, although this was taken into consideration when choosing statistical analyses. As most of the participants who dropped out from treatment could not be assessed and also became study dropouts, no EOT and no follow-up data were available from them. In addition, before the COVID-19 pandemic, patients had in-person intake sessions, including measurements of their weight and height. During the pandemic, the study relied on the patients’ self-reported weight and height. Although BED is more equally prevalent across genders than other eating disorders [75], with only 10% men, the sample was biased by gender. However, no effect of gender was found on eating disorder pathology and the frequency of binges. The underrepresentation of men is common to most eating disorder studies and limits the generalizability of the findings [76]. Finally, therapists’ protocol adherence was measured by self-report of the therapist, whereas the use of an adherence checklist, which recently became available for CBT-E [77], or adherence assessment by an independent rater would have yielded more valid information regarding treatment integrity [78].

### Clinical Implications

Guided self-help CBT-E appears to be an efficacious treatment for patients with BED seeking help from specialized treatment centers. Results of this study underscore the international guidelines following the stepped care model [18] and suggest that web-based guided self-help is a viable first step. If guided self-help CBT-E would appear noninferior to CBT-E, Dutch national guidelines recommending CBT for BED [19] should be revised. In addition, guided self-help CBT-E offers several benefits in delivering psychotherapy to patients with BED, such as reduced barriers to treatment, and if it is noninferior to in-person CBT-E, it will diminish specialist’s time needed for a single treatment. In addition, guided self-help CBT-E has the potential for treatment delivery in a stepped care model to reduce waiting times for in-person treatment [25,29-31]. Furthermore, patients who experience stigma appreciate the greater anonymity of remote treatment [79]. As such, guided self-help CBT-E potentially increases help-seeking behavior among men [80] and patients with excess weight [81]. These benefits of guided self-help CBT-E facilitate treatment delivery, preventing the severity of BED from increasing if left untreated. It is recommended to offer guided self-help CBT-E in specialized settings and experiment with its application in nonspecialist settings. When the findings of this study could be replicated in nonspecialist settings, delivery can be extended to nonspecialist settings. However, supervision of an eating disorder specialist is recommended to address protocol adherence and prevent therapist drift [82].

### Implications for Research

Guided self-help treatment holds promise as a cost-effective alternative to traditional treatments. As an extension of this study, we are currently performing an economic evaluation alongside the RCT (Melisse, B, unpublished data, February 2023). In addition, several studies showed that guided self-help was inferior to in-person CBT at the EOT but was noninferior [20,83] or superior [84] at long-term follow-up. A logical next step for future research is to compare the effectiveness of guided self-help CBT-E with in-person CBT-E in an RCT. We recommend that future studies assess recovery beyond 24 weeks after EOT and collect interview data, as this is deemed more reliable [72]. As guided self-help CBT-E has several additional advantages over traditional treatment provisions, such as reduced therapist time required and removal of geographic barriers to treatment, it is strongly recommended to compare its efficacy with in-person CBT-E. Knowledge of guided self-help predictors or moderators enhances decision-making by offering in-person or guided self-help CBT-E or a different type of treatment [85]. Examining whether guided self-help CBT-E reduces general psychopathology is of interest. Once guided self-help CBT-E shows long-term effectiveness, including general psychopathology, investigating its effect in other eating disorder populations, such as patients with nonpurging bulimia nervosa, is recommended.

### Conclusions

In conclusion, guided self-help CBT-E appeared to be an efficacious treatment alternative to waiting lists regarding reduction in binge eating and eating disorder pathology among patients with BED, and benefits remained over the 12- and 24-week follow-up period. These findings reflect international guidelines recommending guided self-help for BED. If future research would demonstrate equal effectiveness of guided self-help CBT-E to in-person treatment, it would be a viable alternative and can reduce waiting time to commence treatment and, therefore, potentially enhance faster recovery for patients with BED.

## Appendix

Multimedia Appendix 1 CONSORT-eHealth checklist (V1.6.2).

## Abbreviations

BED: binge eating disorder
CBT: cognitive behavioral therapy
CBT-E: cognitive behavioral therapy–enhanced
CIA: clinical impairment assessment
CONSORT: Consolidated Standards of Reporting Trials
CSC: clinically significant change
EDE: Eating Disorder Examination
EDE-Q: Eating Disorder Examination–Questionnaire
EOT: end of treatment
ISO: International Organization for Standardization
OSFED: other specified feeding or eating disorder
RCI: reliable change index
RCT: randomized controlled trial

## Data Availability

Data are available upon reasonable request from author BM.
