# Supplementary material for: Efficacy of Web-Based, Guided Self-help Cognitive Behavioral Therapy–Enhanced for Binge Eating Disorder: Randomized Controlled Trial
Source: J Med Internet Res. 2023 May 1;25:e40472. doi: 10.2196/40472 (PMC10186189; doi:10.2196/40472)
Supplement: Multimedia Appendix 1 [file jmir_v25i1e40472_app1.pdf]

CONSORT-EHEALTH Checklist V1.6.2 Report	Manuscript Number	40472
**(based on CONSORT-EHEALTH V1.6), available at [http://tinyurl.com/consort-ehealth-v1-6].**		
**Date completed**		
4/21/2023 8:10:27		
**by**		
Bernou Melisse		
Efficacy of Web-Based, Guided Self-help Cognitive Behavioral Therapy–Enhanced for Binge Eating Disorder: Randomized Controlled Trial		
TITLE		
**1a-i) Identify the mode of delivery in the title**		
Web-Based, Guided Self-help Cognitive Behavioral Therapy–Enhanced		
**1a-ii) Non-web-based components or important co-interventions in title**		
no, not applicable, the intervention is web based		
**1a-iii) Primary condition or target group in the title**		
' for Binge Eating Disorder'		
ABSTRACT		
**1b-i) Key features/functionalities/components of the intervention and comparator in the METHODS section of the ABSTRACT**		
A single-blind 2-arm randomized controlled trial was designed to evaluate guided self-help CBT-E according to an intention-to-treat analysis. A total of 180 patients were randomly assigned to guided self-help CBT-E (n=90, 50%) or the delayed-treatment control condition (n=90, 50%) for which guided self-help CBT-E was provided after the initial 12-week delay. The primary outcome was reduction in binges. The secondary outcome was full recovery at the end of treatment, as measured using the Eating Disorder Examination during the last 4 weeks of treatment. A linear mixed model analysis was performed to compare treatment outcomes at the end of treatment. A second linear mixed model analysis was performed to measure betweenand within-group effects for up to 24 weeks of follow-up. The Eating Disorder Examination–Questionnaire and clinical impairment assessment were conducted before and after treatment and during follow-up. In addition, dropout rates were assessed in both conditions.'		
**1b-ii) Level of human involvement in the METHODS section of the ABSTRACT**		
' A total of 180 patients were randomly assigned to guided self-help CBT-E (n=90, 50%) or the delayed-treatment control condition (n=90, 50%) ''		
**1b-iii) Open vs. closed, web-based (self-assessment) vs. face-to-face assessments in the METHODS section of the ABSTRACT**		
closed usergroup trial "A total of 180 patients"		
**1b-iv) RESULTS section in abstract must contain use data**		
Of the 180 participants, 142 (78.9%) completed treatment.		
**1b-v) CONCLUSIONS/DISCUSSION in abstract for negative trials**		
Guided self-help CBT-E appeared to be an efficacious treatment'		
INTRODUCTION		
**2a-i) Problem and the type of system/solution**		
Owing to the lack of specialized therapists in the Netherlands, as in many parts of the world, there is a gap between treatment supply and demand [24], resulting in long waiting periods for patients with BED. Therefore, there is an urgent need to increase access to treatment [25]. This situation worsened during the COVID-19 pandemic, when waiting times for treatment increased further and access to care decreased [26]. A remotely offered guided self-help version of CBT-E has the potential to offer treatment with reduced therapist involvement [27]. This, in turn, will enhance treatment availability and thus potentially reduce waiting time before treatment can commence, because long waiting times are unfavorable and associated with a negative treatment outcome [28]'		
**2a-ii) Scientific background, rationale: What is known about the (type of) system**		
Owing to the lack of specialized therapists in the Netherlands, as in many parts of the world, there is a gap between treatment supply and demand [24], resulting in long waiting periods for patients with BED. Therefore, there is an urgent need to increase access to treatment [25]. This situation worsened during the COVID-19 pandemic, when waiting times for treatment increased further and access to care decreased [26]. A remotely offered guided self-help version of CBT-E has the potential to offer treatment with reduced therapist involvement [27]. This, in turn, will enhance treatment availability and thus potentially reduce waiting time before treatment can commence, because long waiting times are unfavorable and associated with a negative treatment outcome [28]'		
**Does your paper address CONSORT subitem 2b?**		
The aim of this study was to examine the efficacy of guided self-help CBT-E compared with that of a delayed-treatment control condition through a randomized controlled trial (RCT) in patients with BED. The primary outcome was reduction in binge eating episodes, and the secondary outcome was the full recovery rate after treatment, as measured during the last 4 weeks of treatment. Web-based, guided self-help CBT-E was hypothesized to be superior to the control condition in reducing binge eating episodes and achieving full recovery. Follow-up measures will be conducted to measure the persistence of treatment benefits. It was hypothesized that treatment gains persist during the 12-week and 24-week follow-up and that there would be no differences between the groups after both groups received treatment.'		
METHODS		
**3a) CONSORT: Description of trial design (such as parallel, factorial) including allocation ratio**		

A superiority RCT to examine the efficacy of web-based, guided self-help CBT-E at end of treatment (EOT) among patients with BED or other specified feeding or eating disorder (OSFED)–BED. Parallel groups were randomly assigned to one of two conditions as follows: (1) guided self-help CBT-E (n=89) or (2) a delayed-treatment control condition (n=91), in which guided self-help CBT-E was offered after a waiting period of 12 weeks. The assessors were blinded to the randomization. In addition, allocation was balanced (1:1) and randomization was stratified for BMI <29.9 kg/m2 or >30 kg/m2 . The guided self-help CBT-E group was assessed at baseline (T0: week 0), week 5 (T1: intermediate evaluation of treatment), week 12 (T2: after treatment), week 24 (T3: 12-week follow-up), and week 36 (T4: 24-week follow-up). The delayed-treatment control group was assessed at baseline (T0: week 0), week 5 (T1: during waiting time), week 12 (T2: start of delayed treatment), week 24 (T3: after treatment), and week 36 (T4: 12-week follow-up). The study was performed in line with the updated CONSORT (Consolidated Standards of Reporting Trials) guidelines for reporting parallel group randomized trials [35].'		
**3b) CONSORT: Important changes to methods after trial commencement (such as eligibility criteria), with reasons**		
As only 10.7% (19/180) of the participants had a BMI <30 kg/m2 , no subgroup analyses based on stratification below and above BMI 30 kg/m2 were performed.'		
**3b-i) Bug fixes, Downtimes, Content Changes**		
this did not happen and is therefore not applicable		
**4a) CONSORT: Eligibility criteria for participants**		
Eligible patients were aged ≥18 years, with a DSM-5 BED or OSFED-BED diagnosis [1], and had a BMI between 19.5 kg/m2 and 40 kg/m2 , because CBT-E was explicitly designed for patients who were not underweight with a BMI of ≤40 kg/m2 [12]. Sufficient proficiency in Dutch and internet access were required. Exclusion criteria were eating disorders other than BED or OSFED-BED, acute psychosis, clinical depression or suicidal ideation, having received eating disorder treatment in the past 6 months, pregnancy, and use of medication that might influence eating behavior. For example, mirtazapine, olanzapine, clozapine, quetiapine, trazodone, and lithium increase appetite, whereas medications including methylphenidate and dexamphetamine decrease appetite [37]. The Dutch version of the semistructured interview the Structured Clinical Interview for DSM-5, Clinician Version (SCID-5-CV), assessing DSM-5 diagnoses [1,38], was used to establish the presence of diagnostic exclusion criteria.''		
**4a-i) Computer / Internet literacy**		
Sufficient proficiency in Dutch and internet access were required. ''		
**4a-ii) Open vs. closed, web-based vs. face-to-face assessments:**		
The interview sections for mood disorders and psychotic disorders were administered. The study was conducted at Novarum, the Dutch Eating Disorders and Obesity Department of Arkin, a large mental health care provider in Amsterdam. All eligible potential participants received verbal and written study information during an advisory session, including an informed consent description, explaining the research goals and information about participation. After patients provided informed consent, a baseline assessment (T0) was scheduled. Recruitment took place between September 2019 and October 2020. Diagnostic interviews were held in person until March 15, 2020, after which, because of the COVID-19 social distancing measures, all interviews were held through videoconferencing'		
**4a-iii) Information giving during recruitment**		
All eligible potential participants received verbal and written study information during an advisory session, including an informed consent description, explaining the research goals and information about participation. '		
**4b) CONSORT: Settings and locations where the data were collected**		
. Interviews were conducted by phone, and self-report measures were administered on the web. All assessments were processed using Castor EDC [49] (International Organization for Standardization [ISO]; ISO 27001/27002/9001 and NEN 7510 certified).''		
**4b-i) Report if outcomes were (self-)assessed through online questionnaires**		
Interview data (EDE) were collected at baseline and after the conclusion of guided self-help CBT-E in the experimental group (T0 and T2). Data from self-report measures (EDE-Q and CIA) were collected at T0, T2, T3, and T4. In addition, the EDE-Q was also completed at T1, 5 weeks after treatment commenced, to evaluate treatment progression between the patient and therapist.'		
**4b-ii) Report how institutional affiliations are displayed**		
no, this is not applicable		
**5) CONSORT: Describe the interventions for each group with sufficient details to allow replication, including how and when they were actually administered**		
**5-i) Mention names, credential, affiliations of the developers, sponsors, and owners**		

Guided Self-help CBT-E Condition<br>Guided self-help CBT-E started in the same week as the baseline assessment. Before commencing treatment, patients were required to read the psychoeducational section of the Dutch version of Overcoming Binge Eating, The Proven Program to Learn Why You Binge and How You Can Stop. Guided self-help CBT-E is a translated and digitalized version of part 2 of the self-help book Overcoming Binge Eating [39]. The intervention ncluded psychoeducation, daily assignments, and 2 self-evaluations each week. When patients did not complete their daily assignments, they received reminders. Patients uploaded their assignments to the web-based therapy environment. Therapists were able to track when the patients logged in, read the psychoeducational parts, and started assignments. Once the patients completed their homework assignments, the therapist received a notification. Subsequently, feedback on the assignments was provided by the therapists during a weekly telephone session of 20 minutes. In the telephone session, completed assignments were discussed, as well as upcoming assignments and compliance with treatment. The sessions were scripted in accordance with the treatment manual as developed by EvdB and BM and offered by therapists through the telephone.<br>Similar to CBT-E–guided self-help, CBT-E consisted of 4 phases; the first stage focused on establishing regular eating and alternatives for binge eating; using real-time self-monitoring as the central intervention; and events, moods, and eating. After a joint review of progress and designing the rest of treatment in the second stage, based on the patients' reported symptoms and maintaining mechanisms of their BED, the third stage focused on either dietary restraint or shape concern and finally ended well with a firm focus on minimizing the risk of relapse in the long term.<br>Delayed-Treatment Control Condition<br>Participants assigned to the delayed-treatment control condition started guided self-help CBT-E 12 weeks after baseline. Thus, their treatment started after a waiting period of the same duration as that of the intervention. Similar to the experimental condition, patients randomized to the control condition were advised to read the psychoeducational section of Overcoming Binge Eating, The Proven Program to Learn Why You Binge and How You Can Stop [39] before commencing treatment. This was recommended to bridge the 12-week waiting period and keep them involved and enrolled in the study. However, these patients did not receive any treatment assignments during this period and did not have access to the web-based treatment environment. Participants were called once after 6 weeks for a short conversation of 10 minutes at most: checking on the eating disorder symptoms and other important areas of life and answering questions about the recommended reading assignment.'		
**5-ii) Describe the history/development process**		
no, this is not reported on, this will be reported elsewhere		
**5-iii) Revisions and updating**		
this was not applicable during the study		
**5-iv) Quality assurance methods**		
To ensure<br>treatment adherence, all therapists attended weekly 45-minute supervision sessions with BM and rated their level of adherence after each session on a scale ranging from 0 (not at all) to 5 (excellent). Self-rated therapist adherence was very good, with 94.7% (1662/1755) of all sessions obtaining a maximum score for excellent adherence.<br>"		
**5-v) Ensure replicability by publishing the source code, and/or providing screenshots/screen-capture video, and/or providing flowcharts of the algorithms used**		
this is not applicable since no algorythms were used		
**5-vi) Digital preservation**		
not applicable, the intervention is yet not open		
**5-vii) Access**		
The study<br>was conducted at Novarum, the Dutch Eating Disorders and Obesity Department of Arkin, a large mental health care provider in Amsterdam. "		
**5-viii) Mode of delivery, features/functionalities/components of the intervention and comparator, and the theoretical framework**		
Patients<br>uploaded their assignments to the web-based therapy environment. Therapists were able to track when the patients logged in, read the psychoeducational parts, and started assignments. Once the patients completed their homework assignments, the therapist received a notification. Subsequently, feedback on the assignments was provided by the therapists during a weekly telephone session of 20 minutes. I"		
**5-ix) Describe use parameters**		
**5-x) Clarify the level of human involvement**		
Treatment was offered by therapists with various backgrounds and educational levels (bachelor's degree for dieticians and nurse practitioners; master's and postdoctoral degree for psychologists). '		
**5-xi) Report any prompts/reminders used**		
When patients did not complete<br>their daily assignments, they received reminders. Patients uploaded their assignments to the web-based therapy environment.'		
**5-xii) Describe any co-interventions (incl. training/support)**		
there were no co inerventions		
**6a) CONSORT: Completely defined pre-specified primary and secondary outcome measures, including how and when they were assessed**		

The guided self-help CBT-E group was assessed at baseline (T0: week 0), week 5 (T1: intermediate evaluation of treatment), week 12 (T2: after treatment), week 24 (T3: 12-week follow-up), and week 36 (T4: 24-week follow-up). The delayed-treatment control group was assessed at baseline (T0: week 0), week 5 (T1: during waiting time), week 12 (T2: start of delayed treatment), week 24 (T3: after treatment), and week 36 (T4: 12-week follow-up)' and 'The primary outcome indicator was reduction in binge eating at T2. Binge eating was measured during the last 28 days using the Dutch Eating Disorder Examination (EDE), a validated expert interview tool. The secondary outcome indicator was full recovery at T2, which was defined as an EDE global score <1.77 as well as abstinence from binge eating during the last 28 days [40]. The cutoff on the EDE global score of <1.77 was based on the community mean plus 1 SD [41,42]. Other outcome measures were reliable change index (RCI) and clinically significant change (CSC) [43,44]. RCI was established as RCI=0.54 on the EDE global score, and CSC was defined as EDE global score <1.77 as well as a pre- to posttest change >RCI [41,43]. Outcome measures on self-report data were reduction of binge eating during the last 4 weeks measured at T2, T3, and T4 with the Dutch version of the EDE-Questionnaire (EDE-Q), a validated self-report questionnaire [45,46]. Full recovery was defined as an EDE-Q global score <2.77 (based on the community mean plus 1 SD) combined with the absence of binges, as described in Turner et al [40,47,48]. Cutoff on the EDE-Q was 2.77 and RCI was 0.63 on the EDE-Q global score, together they defined CSC [43,45]. The last outcome measure was the reduction of secondary impairment from eating disorder behavior during the last 28 days, as measured by the clinical impairment assessment (CIA) [5]. Interview data (EDE) were collected at baseline and after the conclusion of guided self-help CBT-E in the experimental group (T0 and T2). Data from self-report measures (EDE-Q and CIA) were collected at T0, T2, T3, and T4. In addition, the EDE-Q was also completed at T1, 5 weeks after treatment commenced, to evaluate treatment progression between the patient and therapist'.

6a-i) Online questionnaires: describe if they were validated for online use and apply CHERRIES items to describe how the questionnaires were designed/deployed

6a-ii) Describe whether and how "use" (including intensity of use/dosage) was defined/measured/monitored

6a-iii) Describe whether, how, and when qualitative feedback from participants was obtained

6b) CONSORT: Any changes to trial outcomes after the trial commenced, with reasons

. Interviews were conducted by phone, and self-report measures were administered on the web. All assessments were processed using Castor EDC [49] (International Organization for Standardization [ISO]; ISO 27001/27002/9001 and NEN 7510 certified)."

7a) CONSORT: How sample size was determined

7a-i) Describe whether and how expected attrition was taken into account when calculating the sample size

On the basis of other self-help interventions, a 46% decrease in binge eating behavior was expected over time [22]. The expected effect size was a Cohen d of 0.47 between the experimental and control conditions [22,50]. To achieve sufficient power (β=.8), the required sample size was 144 (n=72 per arm). As a 20% dropout was estimated [22], more participants were included: N=180 (n=90 per arm), resulting in n=72 expected completers, yielding a power of β=.8, with an effect size of Cohen d=0.47, at α=.05 (2-sided). Sample size was calculated using R package (R Foundation for Statistical Computing) pwr [51].'.

7b) CONSORT: When applicable, explanation of any interim analyses and stopping guidelines

The guided self-help CBT-E group was assessed at baseline (T0: week 0), week 5 (T1: intermediate evaluation of treatment), week 12 (T2: after treatment), week 24 (T3: 12-week follow-up), and week 36 (T4: 24-week follow-up). The delayed-treatment control group was assessed at baseline (T0: week 0), week 5 (T1: during waiting time), week 12 (T2: start of delayed treatment), week 24 (T3: after treatment), and week 36 (T4: 12-week follow-up)' and 'The primary outcome indicator was reduction in binge eating at T2. Binge eating was measured during the last 28 days using the Dutch Eating Disorder Examination (EDE), a validated expert interview tool. The secondary outcome indicator was full recovery at T2, which was defined as an EDE global score <1.77 as well as abstinence from binge eating during the last 28 days [40]. The cutoff on the EDE global score of <1.77 was based on the community mean plus 1 SD [41,42]. Other outcome measures were reliable change index (RCI) and clinically significant change (CSC) [43,44]. RCI was established as RCI=0.54 on the EDE global score, and CSC was defined as EDE global score <1.77 as well as a pre- to posttest change >RCI [41,43]. Outcome measures on self-report data were reduction of binge eating during the last 4 weeks measured at T2, T3, and T4 with the Dutch version of the EDE-Questionnaire (EDE-Q), a validated self-report questionnaire [45,46]. Full recovery was defined as an EDE-Q global score <2.77 (based on the community mean plus 1 SD) combined with the absence of binges, as described in Turner et al [40,47,48]. Cutoff on the EDE-Q was 2.77 and RCI was 0.63 on the EDE-Q global score, together they defined CSC [43,45]. The last outcome measure was the reduction of secondary impairment from eating disorder behavior during the last 28 days, as measured by the clinical impairment assessment (CIA) [5]. Interview data (EDE) were collected at baseline and after the conclusion of guided self-help CBT-E in the experimental group (T0 and T2). Data from self-report measures (EDE-Q and CIA) were collected at T0, T2, T3, and T4. In addition, the EDE-Q was also completed at T1, 5 weeks after treatment commenced, to evaluate treatment progression between the patient and therapist'.

8a) CONSORT: Method used to generate the random allocation sequence

The assessors were blinded to the randomization. In addition, allocation was balanced (1:1) and randomization was stratified for BMI <29.9 kg/m2
or >30 kg/m2
.'

8b) CONSORT: Type of randomisation; details of any restriction (such as blocking and block size)

The assessors were blinded to the randomization. In<br>addition, allocation was balanced (1:1) and randomization was<br>stratified for BMI <29.9 kg/m2<br>or >30 kg/m2<br>."		
**9) CONSORT: Mechanism used to implement the random allocation sequence (such as sequentially numbered containers), describing any steps taken to conceal the sequence until interventions were assigned**		
The assessors were blinded to the randomization. In<br>addition, allocation was balanced (1:1) and randomization was<br>stratified for BMI <29.9 kg/m2<br>or >30 kg/m2<br>.'		
**10) CONSORT: Who generated the random allocation sequence, who enrolled participants, and who assigned participants to interventions**		
Randomizations were performed by administrative staff<br>members of another department in Castor EDC [49] by a 4, 6,<br>8 block design. '		
**11a) CONSORT: Blinding - If done, who was blinded after assignment to interventions (for example, participants, care providers, those assessing outcomes) and how**		
**11a-i) Specify who was blinded, and who wasn't**		
In addition, when offering treatment, therapists<br>were not aware of whether patients had previously been<br>allocated to the experimental or control condition.' and 'The assessors were blinded to the randomization.'		
**11a-ii) Discuss e.g., whether participants knew which intervention was the "intervention of interest" and which one was the "comparator"**		
**11b) CONSORT: If relevant, description of the similarity of interventions**		
there were no similarities in the interventions		
**12a) CONSORT: Statistical methods used to compare groups for primary and secondary outcomes**		
Baseline Differences<br>The significance of baseline differences between the groups<br>was examined using chi-square tests or ANOVA.<br>Treatment Adherence<br>Regression analyses were conducted to assess whether baseline<br>scores (number of objective binges, eating disorder severity,<br>and BMI) and demographics (age, gender, level of education,<br>profession, and country of birth) predicted treatment completion.<br>2 × 2 Design<br>The primary outcome was treatment effects based on interview<br>data (EDE) with regard to reduction in binge eating episodes<br>and full recovery at posttest between the experimental and delayed-treatment control group, which were compared after<br>12 weeks, when the experimental group had concluded treatment<br>(T2). As patients were initially supposed to be nested within<br>their BMI group as described in the protocol [36], for the<br>primary outcome measures, a 2 × 2 design was used using a<br>generalized linear mixed model analysis [52], with group as the<br>between-subjects factor and time of assessment as the<br>within-subjects factor at the primary end point. As full recovery<br>was a binary variable, a negative binomial model with log link<br>was used.<br>2 × 5 Design<br>Self-report data (EDE-Q and CIA) were analyzed with a 2 × 5<br>generalized linear mixed model analysis [52], with group as the<br>between-subjects factor and time of assessment as the<br>within-subjects factor, which also measured persistence of<br>treatment benefits after EOT. For full recovery (binary variable),<br>we used a negative binomial model with log link.'.		
**12a-i) Imputation techniques to deal with attrition / missing values**		
Analyses were performed according to an intention-to-treat<br>approach (imputed data set with 25 imputations for each missing<br>observation) [53]. Imputations were performed with the multiple<br>imputation by chained equations, using predictive mean<br>matching combining 25 imputations in R package mice [54].<br>All other statistical analyses were performed using SPSS (IBM<br>Corp) versions 25 and 28.'		
**12b) CONSORT: Methods for additional analyses, such as subgroup analyses and adjusted analyses**		
yes, this is described in the above items		
RESULTS		
**13a) CONSORT: For each group, the numbers of participants who were randomly assigned, received intended treatment, and were analysed for the primary outcome**		
this is addressed in the flowchart (figure 1)		
**13b) CONSORT: For each group, losses and exclusions after randomisation, together with reasons**		
this is addressed in the flowchart (figure 1)		
**13b-i) Attrition diagram**		
**14a) CONSORT: Dates defining the periods of recruitment and follow-up**		
Recruitment took place between September<br>2019 and October 2020.'		
**14a-i) Indicate if critical "secular events" fell into the study period**		
**14b) CONSORT: Why the trial ended or was stopped (early)**		
the trial was not stopped early but when the number of pp were recruited		
**15) CONSORT: A table showing baseline demographic and clinical characteristics for each group**		
this is described in table 1		
**15-i) Report demographics associated with digital divide issues**		
this is described in table 1		
**16a) CONSORT: For each group, number of participants (denominator) included in each analysis and whether the analysis was by original assigned groups**		
**16-i) Report multiple "denominators" and provide definitions**		
this is described in table 2		
**16-ii) Primary analysis should be intent-to-treat**		
There were no differences between the<br>intention-to-treat and the completers sample.<br>Table 2. Changes in binge eating behaviors and Eating Disorder Examination (EDE) scores over the course of treatment assessed using intention-to-treat analysis with multiple imputations'		
**17a) CONSORT: For each primary and secondary outcome, results for each group, and the estimated effect size and its precision (such as 95% confidence interval)**		
yes, this is described in table 2		
**17a-i) Presentation of process outcomes such as metrics of use and intensity of use**		
**17b) CONSORT: For binary outcomes, presentation of both absolute and relative effect sizes is recommended**		
they are described in table 2		
**18) CONSORT: Results of any other analyses performed, including subgroup analyses and adjusted analyses, distinguishing pre-specified from exploratory**		
this is not applicable, no changes were made		

18-i) Subgroup analysis of comparing only users

19) CONSORT: All important harms or unintended effects in each group

no harms appeared and no SAE

19-i) Include privacy breaches, technical problems

19-ii) Include qualitative feedback from participants or observations from staff/researchers

DISCUSSION

20) CONSORT: Trial limitations, addressing sources of potential bias, imprecision, multiplicity of analyses

20-i) Typical limitations in ehealth trials

Strengths and Limitations
This study has several strengths. It was conducted in a specialized mental health care setting, acknowledged for its highly structured treatment and evidence-based approach. Guided self-help CBT-E was a manualized treatment offered by trained specialists and treatment adherence was assessed. Standardized interview data [71] were collected by independent assessors, including the EDE at T2. Internationally used valid self-report instruments [5,45] were used, and the study was adequately powered. As patients came from all over the Netherlands, the sample can be deemed representative of patients seeking specialized eating disorder treatment. The COVID-19 pandemic deserves a special mention. The study barely started when the COVID-19 pandemic spread in the Netherlands in mid-March 2020. Fortunately, however, because of the treatment delivery mode (e–mental health) that was evaluated in this study, the social distancing measures of the pandemic had a limited impact on the study's execution. Nevertheless, the COVID-19 pandemic might have negatively affected the outcomes of the treatments, as many patients reported that it was a challenge to combine therapy, work, and homeschooling children at the same time. This suggests that guided self-help CBT-E might demonstrate even better outcomes under less adverse circumstances.
A limitation of this study might be that the follow-up data were measured by self-report, and interview data are generally viewed as more reliable, especially when measuring binge eating behavior [72,73]. In addition, our study showed differences in reports on interviews and self-report data. Objective binges between the interview and self-report data in this study showed a moderate correlation (r=0.6; P<.001) at T2. The study's design with a delayed-treatment control group implies that expected treatment benefits may have played a role in bringing about the difference in outcomes at the second assessment [74]. However, the extent of this effect could not be established, as treatment expectancy was not assessed. Next, between-group comparisons were impacted as the control group started treatment after the 12-week delay. Therefore, the long-term impact of withholding treatment could not be assessed. The control group showed a delayed treatment effect very similar to that of the guided self-help group, consistent with the delayed design. Furthermore, only within-group comparisons were meaningful during follow-up, although this was taken into consideration when choosing statistical analyses. As most of the participants who dropped out from treatment could not be assessed and also became study dropouts, no EOT and no follow-up data were available from them. In addition, before the COVID-19 pandemic, patients had in-person intake sessions, including measurements of their weight and height. During the pandemic, the study relied on the patients'self-reported weight and height. Although BED is more equally prevalent across genders than other eating disorders [75], with only 10% men, the sample was biased by gender. However, no effect of gender was found on eating disorder pathology and the frequency of binges. The underrepresentation of men is common to most eating disorder studies and limits the generalizability of the findings [76].
Finally, therapists' protocol adherence was measured by self-report of the therapist, whereas the use of an adherence checklist, which recently became available for CBT-E [77], or adherence assessment by an independent rater would have yielded more valid information regarding treatment integrity [78]'

21) CONSORT: Generalisability (external validity, applicability) of the trial findings

21-i) Generalizability to other populations

As patients came from all over the Netherlands, the sample can be deemed representative of patients seeking specialized eating disorder treatment"

21-ii) Discuss if there were elements in the RCT that would be different in a routine application setting

22) CONSORT: Interpretation consistent with results, balancing benefits and harms, and considering other relevant evidence

22-i) Restate study questions and summarize the answers suggested by the data, starting with primary outcomes and process outcomes (use)

The aim of this study was to examine the efficacy of guided self-help CBT-E compared with a delayed-treatment control group regarding reduction in objective binges. The efficacy of guided self-help CBT-E was demonstrated by its superiority in outcome over the delayed-treatment control condition at T2.
On the basis of reduction in binge eating, a large effect size (Cohen d=1.0) was observed. Binge eating reduced from an average of 19 objective binges 28 days before assessment to 3 binges after completion of guided self-help CBT-E, compared with 16 to 13 binges in the control group. In the guided self-help condition, abstinence from binge eating at T2 was reported by 48% (43/90) of the participants according to the EDE interview."

22-ii) Highlight unanswered new questions, suggest future research

Other information

23) CONSORT: Registration number and name of trial registry

NTR 7994; https://trialregister.nl/trial/7994"

24) CONSORT: Where the full trial protocol can be accessed, if available

RR2-10.1186/s12888-020-02604-1"

25) CONSORT: Sources of funding and other support (such as supply of drugs), role of funders

no fumding was received for this study

X26-i) Comment on ethics committee approval

Study approval (reference number NL 6958.100.19) was granted in August 2019 by the Medical Research Ethics Committees United in Nieuwegein, the Netherlands. All patients were informed about the study and assured that their data were deidentified, and all patients signed an informed consent form.'		
**x26-ii) Outline informed consent procedures**		
**X26-iii) Safety and security procedures**		
**X27-i) State the relation of the study team towards the system being evaluated**		
Conflicts of Interest<br>None declared"		
